# Eleven strategies for making reproducible research and open science training the norm at research institutions

**DOI:** 10.7554/eLife.89736

**Published:** 2023-11-23

**Authors:** Friederike E Kohrs, Susann Auer, Alexandra Bannach-Brown, Susann Fiedler, Tamarinde Laura Haven, Verena Heise, Constance Holman, Flavio Azevedo, René Bernard, Arnim Bleier, Nicole Bössel, Brian Patrick Cahill, Leyla Jael Castro, Adrian Ehrenhofer, Kristina Eichel, Maximillian Frank, Claudia Frick, Malte Friese, Anne Gärtner, Kerstin Gierend, David Joachim Grüning, Lena Hahn, Maren Hülsemann, Malika Ihle, Sabrina Illius, Laura König, Matthias König, Louisa Kulke, Anton Kutlin, Fritjof Lammers, David MA Mehler, Christoph Miehl, Anett Müller-Alcazar, Claudia Neuendorf, Helen Niemeyer, Florian Pargent, Aaron Peikert, Christina U Pfeuffer, Robert Reinecke, Jan Philipp Röer, Jessica L Rohmann, Alfredo Sánchez-Tójar, Stefan Scherbaum, Elena Sixtus, Lisa Spitzer, Vera Maren Straßburger, Marcel Weber, Clarissa J Whitmire, Josephine Zerna, Dilara Zorbek, Philipp Zumstein, Tracey L Weissgerber

**Affiliations:** 1 QUEST Center for Responsible Research, Berlin Institute of Health at Charité - Universitätsmedizin Berlin Berlin Germany; 2 Department of Plant Physiology, Faculty of Biology, Technische Universität Dresden Dresden Germany; 3 Department Strategy & Innovation, Vienna University of Economics and Business Vienna Austria; 4 Danish Centre for Studies in Research & Research Policy, Department of Political Science, Aarhus University Aarhus Denmark; 5 Freelance researcher Gladbeck Germany; 6 Saxony Center for Criminological Research Chemnitz Germany; 7 University of Cambridge Cambridge United Kingdom; 8 NeuroCure Cluster of Excellence, Charité - Universitätsmedizin Berlin Berlin Germany; 9 Department for Computational Social Sciences, GESIS - Leibniz Institute for the Social Sciences Cologne Germany; 10 Department of Psychiatry and Psychotherapy, University Medicine Greifswald Greifswald Germany; 11 Leibniz Information Centre for Science and Technology Hannover Germany; 12 ZB MED Information Centre for Life Sciences Cologne Germany; 13 Institute of Solid Mechanics & Dresden Center for Intelligent Materials, Technische Universität Dresden Dresden Germany; 14 Department of Education and Psychology, Freie Universität Berlin Berlin Germany; 15 Department Psychology, LMU Munich Munich Germany; 16 Institute of Information Science, Technische Hochschule Köln Köln Germany; 17 Department of Psychology, Saarland University Saarbrücken Germany; 18 Department of Psychology, Technische Universität Dresden Dresden Germany; 19 Department of Biomedical Informatics at the Center for Preventive Medicine and Digital Health, Medical Faculty Mannheim, Heidelberg University Heidelberg Germany; 20 Department of Psychology, Heidelberg University Heidelberg Germany; 21 Department of Survey Development and Methodology, GESIS – Leibniz Institute for the Social Sciences Mannheim Germany; 22 Department of Social Psychology, Universität Trier Trier Germany; 23 LMU Open Science Center, Department of Psychology, LMU Munich Munich Germany; 24 ICAN Institute for Cognitive and Affective Neuroscience, Department of Psychology, Faculty of Human Sciences, Medical School Hamburg Hamburg Germany; 25 Faculty of Life Sciences: Food, Nutrition and Health, University of Bayreuth Bayreuth Germany; 26 Institute for Biology, Institute for Theoretical Biology, Humboldt-University Berlin Berlin Germany; 27 Developmental Psychology with Educational Psychology, University of Bremen Bremen Germany; 28 Max Planck Institute for the Physics of Complex Systems Dresden Germany; 29 Division of Regulatory Genomics and Cancer Evolution, German Cancer Research Center (DKFZ) Heidelberg Germany; 30 Department of Psychiatry, Psychotherapy and Psychosomatics, Medical School, RWTH Aachen University Aachen Germany; 31 Computation in Neural Circuits, Max Planck Institute for Brain Research Frankfurt Germany; 32 Hector-Institute for Education Sciences and Psychology, Eberhard Karls, University of Tübingen Tübingen Germany; 33 Department of Education and Psychology, Freie Universität Berlin Berlin Germany; 34 Center for Lifespan Psychology, Max Planck Institute for Human Development Berlin Germany; 35 Department of Psychology, Catholic University of Eichstätt-Ingolstadt Eichstätt Germany; 36 Institute of Geography, Johannes Gutenberg-University Mainz Mainz Germany; 37 Department of Psychology and Psychotherapy, Witten/Herdecke University Witten Germany; 38 Scientific Directorate, Max Delbrück Center for Molecular Medicine in the Helmholtz Association (MDC) Berlin Germany; 39 Department of Evolutionary Biology, Bielefeld University Bielefeld Germany; 40 Empirical Childhood Research, University of Potsdam Potsdam Germany; 41 Leibniz Institute for Psychology Trier Germany; 42 Department of Psychology, Medical School Hamburg Hamburg Germany; 43 Charité - Universitätsmedizin Berlin, Gender in Medicine (GiM) Berlin Germany; 44 Max Delbrück Center for Molecular Medicine in the Helmholtz Association Berlin Germany; 45 Neuroscience Research Center, Charité-Universitätsmedizin Berlin Berlin Germany; 46 International Graduate Program Medical Neurosciences, Charité – Universitätsmedizin Berlin Berlin Germany; 47 Open Science Office, University of Mannheim Mannheim Germany; Icahn School of Medicine at Mount Sinai United States; Icahn School of Medicine at Mount Sinai United States

**Keywords:** reproducible research, scientific rigor, transparency, open science, higher education, curriculum design

## Abstract

Reproducible research and open science practices have the potential to accelerate scientific progress by allowing others to reuse research outputs, and by promoting rigorous research that is more likely to yield trustworthy results. However, these practices are uncommon in many fields, so there is a clear need for training that helps and encourages researchers to integrate reproducible research and open science practices into their daily work. Here, we outline eleven strategies for making training in these practices the norm at research institutions. The strategies, which emerged from a virtual brainstorming event organized in collaboration with the German Reproducibility Network, are concentrated in three areas: (i) adapting research assessment criteria and program requirements; (ii) training; (iii) building communities. We provide a brief overview of each strategy, offer tips for implementation, and provide links to resources. We also highlight the importance of allocating resources and monitoring impact. Our goal is to encourage researchers – in their roles as scientists, supervisors, mentors, instructors, and members of curriculum, hiring or evaluation committees – to think creatively about the many ways they can promote reproducible research and open science practices in their institutions.

## Introduction

In recent years, awareness of the importance of reproducible research and open science has grown in the research community. The importance of conducting robust, transparent, and open research has especially been highlighted by the reproducibility crisis, or credibility revolution (Baker, 2016; Errington et al., 2021; Vazire, 2018). Reproducible and open science practices increase the likelihood that research will yield trustworthy results, and facilitate reuse of methods, data, code, and software (Chan et al., 2014; Diaba-Nuhoho and Amponsah-Offeh, 2021; Downs, 2021; Ioannidis et al., 2014). Across fields, definitions of ‘reproducible’ and ‘open’ may vary. While some fields use the terms interchangeably, in other fields ‘reproducible’ includes elements of scientific rigor and research quality, whereas ‘open’ simply refers to making research outputs publicly accessible. Overall, these practices seek to improve the transparency, trustworthiness, reusability, and accessibility of scientific findings for the research community and society (Barba, 2018; Claerbout and Karrenbach, 1992; Nosek et al., 2022; Parsons et al., 2022; Wolf, 2017). Examples of specific practices include sharing of protocols, data and code, publishing open access, implementing practices such as blinding and randomization to reduce the risk of bias, engaging patients in designing and conducting research, using reporting guidelines to improve reporting, and using CRediT authorship statements to specify author contributions. Despite these developments, reproducible research and open science practices remain uncommon in many fields (Blanco et al., 2019; Grant et al., 2013; Hardwicke et al., 2022; Hardwicke et al., 2020; Page and Moher, 2017).

According to a survey by the European University Association (EUA) for 2020–2021, 59% of the 272 European institutions surveyed rated open science’s strategic importance at the institutional level as very high or high (Morais et al., 2021). The strategic importance of open science has also been recognized by policy-makers, for example by the UNESCO Recommendations on Open Science (UNESCO, 2021). Unfortunately, these values are not reflected in the current research assessment system. ‘Classic’ research assessment criteria, such as the Journal Impact Factor or the h-index, are still being used to assess the contribution of individual researchers. The use of these biased metrics should be discouraged, however, as they ignore the value of other research outputs (e.g. protocols, data, code) and are not useful for assessing the impact and quality of individual research contributions (https://sfdora.org/read/). Initiatives such as COARA seek to reform research assessment criteria to recognize a broad range of activities that contribute to high quality research (https://coara.eu/). These reforms are essential to incentivize reproducible research and open science practices.

In addition to shifting incentives, effective education and training programs that teach reproducible research and open science skills have not yet been implemented across research fields. Researchers in various disciplines are discussing whether these concepts apply, and how they might be implemented. To explore these ideas, German Reproducibility Network members organized a virtual brainstorming event (see Box 1) to discuss strategies for making reproducible research and open science training the norm at research institutions in Germany and beyond.

Box 1. **Virtual unconference format**

In March 2022, 96 participants, consisting mostly of members of initiatives and organizations belonging to the German Reproducibility Network (GRN) and other researchers based in Germany, took part in the virtual brainstorming event. Participants came from a variety of professional backgrounds (e.g. academic researchers, administrators, library and information science professionals), career stages (from graduate students to senior group leaders), and disciplines (e.g. psychology, biomedical sciences). The virtual brainstorming event unconference format has been explained previously (Holman et al., 2021). Supplementary file 1 provides details of this specific event. This paper shares lessons learned from two days of intensive discussions, through virtual networking events, virtual meetings, and asynchronous conversations on an online discussion board.

The first section of this paper provides a brief overview of eleven strategies that were derived from the event. Members of the research community can implement these strategies by taking action in their roles as instructors, researchers, supervisors, mentors, members of curriculum or hiring and evaluation committees, or as part of institutional leadership, research support or administrative teams. The section also highlights actions that institutions can take to support these activities, by allocating resources and monitoring impact. The second section of this paper lists a few tips for implementing several strategies. Cited resources provide additional insights for those interested in pursuing specific strategies. While making reproducible and open science training the norm might involve major changes at institutions, this journey starts with small steps towards reproducible and open science practices. Changing norms will require a broad coalition; hence, we hope that this piece inspires others to join this effort, while encouraging those who are already engaged to think creatively about opportunities to enhance the impact of their work.

## The eleven strategies

**Figure 1.**
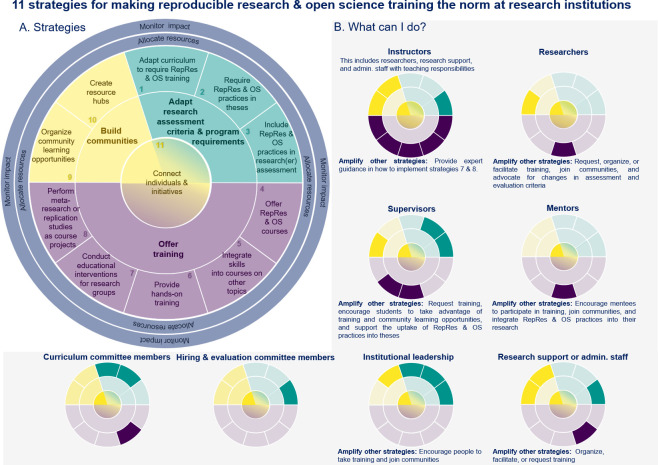
Eleven strategies for making reproducible research & open science training the norm at research institutions. The eleven strategies are concentrated in three areas: (1) adapting research assessment criteria and program requirements (cyan), (2) offering training (purple), and (3) building communities (yellow). While Strategy 11 is part of the ‘build communities’ category, it is placed at the center to highlight the importance of building connections with others working on strategies in other areas. Institutions can support those working on the eleven strategies by allocating resources and monitoring impact. These activities are shown as two blue rings encircling the eleven strategies. (**A**). The small multiples (small versions of the main graph) highlight the strategies that different stakeholders can directly use at their institutions. The text below describes opportunities for different stakeholder groups to amplify or support the efforts of those working on other strategies (**B**). While the roles are briefly defined below, these general definitions may vary by country, field or institution. The figure provides a high-level overview; however, the strategies that are most relevant to a particular individual may diverge from what is shown depending on his or her specific responsibilities and activities. Many individuals fulfill multiple roles. **Definition of roles: Instructors** include researchers and other staff who teach courses or provide hands-on training. **Researchers** include more established scientists, early career researchers (ECRs), research trainees and others who design and conduct research studies. **Supervisors** provide guidance and advice on the student’s research activities, but also take part in the examination and evaluation of the student’s progress and performance. **Mentors** support the career development of less experienced researchers by meeting regularly with mentees to share advice, perspectives, and skills. **Curriculum committee members** serve on committees that design and/or approve curriculum for degree programs. **Hiring and evaluation committee members** serve on committees that hire, assess or promote researchers. **Institutional leadership** includes those in high-level positions who set priorities and establish policies for the institution (e.g. dean, provost, department chair). **Research support or administrative staff** may include librarians, information technology professionals, data stewards, core facility staff, open science officers, staff working with regulatory committees (e.g., ethics committees or institutional animal care and use committees), and others who support researchers. Abbreviations: RepRes, reproducible research; OS, open science.

The eleven strategies derived from the event discussions fall into three categories: (i) adapting research assessment criteria and program requirements [strategies 1–3]; (ii) training [strategies 4–8]; (iii) building communities [strategies 9–11]. Figure 1 illustrates these strategies, and highlights stakeholder groups that can directly contribute to each strategy or amplify the efforts of others. The stakeholder groups examined include instructors, researchers, supervisors, mentors, members of curriculum or hiring and evaluation committees, institutional leadership, and research administration and support staff. Research institutions can further support those working on these eleven strategies by allocating resources and monitoring impact for ongoing activities.

### Adapt research assessment criteria and program requirements

#### Strategy 1: Adapt curriculum to require reproducibility and open science training

Required courses reach more students than elective courses; hence, integrating courses into a required curriculum is an important step towards making reproducibility and open science training the norm. This could include adding or expanding research methods courses to cover topics such as protocol depositing, open data and code, and rigorous experimental design.

One example for undergraduate students is the Munich core curriculum for empirical practice courses. This requires that topics such as sample size planning and power analysis, preregistration, open data, and reproducible analysis scripts be included in all empirical practice courses in the Bachelors curriculum at the Department of Psychology at LMU Munich (Schönbrodt et al., 2022; Schönbrodt et al., 2015). At Goethe University Frankfurt am Main, courses on research methods and evaluation were included in Psychology Master’s programs to teach basic statistical skills and more advanced topics, such as selection bias and meta-analysis.

For graduate students, open and reproducible research practices may also be incorporated into existing required training on research integrity or good scientific/clinical practice. For example, PhD candidates may be required to attend a good scientific practice course prior to finishing their work (e.g. at the Faculty of Psychology at the Technische Universität Dresden). Sometimes these courses only cover research misconduct; it is important that courses also address reproducible research and open science practices. Collaborative Research Centers funded by the Deutsche Forschungsgemeinschaft (German Research Foundation, DFG) may require PhD students to attend workshops on good scientific practice, research transparency and research data management. This training can be accompanied by locally organized lectures and meetings on open science (e.g., at the CRC 940 of the Technische Universität Dresden).

#### Strategy 2: Require reproducible research and open science practices in undergraduate and graduate theses

Degree programs may require reproducible research and open science practices in undergraduate or graduate theses. Requirements will depend on the field and program, as illustrated in the examples below.

In Germany, psychology departments have taken on a leading role in implementing this strategy. Many departments (e.g. Department of Social Psychology at Trier University, Department of Psychology at Saarland University, Faculty of Psychology at Technische Universität Dresden) already include guidelines or guiding principles on quality assurance and open science practices in thesis agreements for Bachelor’s and Master’s programs.

Reproducible research and open science practices have also been included in PhD thesis agreements. For example, the Department of Psychology at LMU Munich requires PhD students and their primary supervisors to agree on a list of planned open science practices (e.g. open access, open data, or preregistration) before starting the thesis. All implemented practices are described in a disclosure form, which is submitted with the completed PhD thesis. PhD students in the Department of Psychology and Sport Science at the University of Münster need to submit a similar disclosure form with their thesis.

An alternative approach is to encourage students tnduct replication studies, evidence synthesis, or meta-research as part of graduate theses. In epidemiology, for example, students routinely conduct a systematic literature review as part of their PhD. Graduate programs that adopt this approach need to recognize these types of studies as fulfilling graduation requirements.

#### Strategy 3: Include reproducible and open science practices in research(er) assessment

Traditional assessment criteria for hiring and evaluation of individual researchers still focus on third-party funding and the number of publications. Unfortunately, these criteria do not incentivize or reward reproducible research and open science practices. Furthermore, this approach can encourage researchers to publish more at the expense of research quality (Abele-Brehm and Bühner, 2016; Allen and Mehler, 2019; Smaldino and McElreath, 2016). A growing number of coalitions and initiatives are under way to reform the way we assess research(ers) (e.g. CoARA: https://coara.eu/about/, DORA: https://sfdora.org/read/, some activities within LERU: https://www.leru.org/, European Commission Directorate General for Research and Innovation, 2021).

Some institutions and departments have begun incorporating reproducible and open science practices in hiring and evaluation processes (Lingervelder et al., 2022; Kip and Dirnagl, 2021; Pontika et al., 2022; Schönbrodt, 2019; HI-FRAME, University of Zurich). The growing list of academic job offers that mention open science contributions (Schönbrodt et al., 2018) suggests that research(er) assessment practices are beginning to change. However, only a few institutions have released official policies on the inclusion of reproducible and open science requirements in academic job descriptions and hiring processes. For instance, the Department of Psychology at LMU Munich asks professorship applicants to include a statement on how they have already implemented and plan to further implement open science practices (Schönbrot, 2016). There are concrete proposals on how to implement responsible research assessment, such as establishing a minimum methodological rigor threshold that candidates need to pass in order to be considered for hiring and promotion (Gärtner et al., 2022; Schönbrodt et al., 2022).

### Offer training

#### Strategy 4: Offer reproducible research and open science courses

This was the most common activity that event participants engaged in. Formats included single lectures, webinar series, workshops (which can range from half a day to several days), summer schools, and courses (Grüning and Frank, 2023). While training was occasionally integrated into undergraduate or graduate curriculum requirements (see strategies 6 and 7), most courses were electives, often run by early career researchers (ECRs) for other ECRs. Some instructors offered field-specific training, while others addressed multidisciplinary audiences.

Many pre-existing examples of this format are open access (e.g. repro4everyone; Auer et al., 2021; Boyle et al., 2023); therefore, we encourage readers to search for examples that are relevant to the course formats and topics that interest them.

#### Strategy 5: Integrate reproducibility and open science skills into courses on other topics

Even when reproducible and open research skills are not part of the official curricula, instructors who teach required courses on other topics can integrate reproducible research and open science skills. This might include giving a lecture on the implications of the reproducibility crisis and potential solutions in an introductory class, integrating pre-registrations into research project courses, using open science tools to analyze and present data during undergraduate practical training, or practicing techniques for writing reproducible protocols in laboratory sessions. In seminars, participants can critically debate whether selected publications fulfill necessary quality standards.

One example is the peer review training course Peerspectives (Rohmann et al., 2022), which integrates reproducibility and open science topics by encouraging students to examine and comment on which reproducible research and open research practices were applied in the papers that students peer review.

#### Strategy 6: Provide hands-on training

Traditional courses and workshops often cover many practices in a short time; hence, participants need to decide which practices to implement, and how to implement them, after returning to their research group (Heise et al., 2023). In contrast, participants in hands-on courses implement practices in their own research during training. After completing training, students have direct evidence that they have implemented what they learned.

One example is ReproducibiliTeach, a flipped course where participants watch brief videos introducing reproducible research practices prior to class. During class, participants directly implement what they have learned in their own research. Book dashes and hackathons, such as those organized by the Turing Way and ReproHack respectively, also provide hands-on training. These can be offered as standalone events or integrated into traditional courses (https://www.reprohack.org/, https://the-turing-way.netlify.app/community-handbook/bookdash.html).

#### Strategy 7: Conduct educational interventions for research groups

Implementing reproducible research and open science practices often requires collaboration among members of a research team. Researchers who completed a course independently may have difficulties convincing other members of their research team to invest time and resources into learning and adopting new practices (Heise et al., 2023). In contrast, interventions designed for research groups may facilitate change by ensuring that all team members receive the same training and can collaboratively implement new practices.

For example, research groups can incorporate open data practices into their everyday research routines by completing a multi-week intervention that includes regular group meetings and a reading list (Lowndes et al., 2019).

#### Strategy 8: Perform replication or meta-research studies as course projects

Rather than teaching reproducible research or open science skills that researchers can use in their project (e.g. use of reporting guidelines, open data), this approach trains participants to conduct meta-research (science of science) or replication studies. As the class collaborates on one project, participants also build skills for collaborative team science and gain experience leading small teams. Examples include conducting a replication study with students to teach longitudinal data analysis techniques (Seibold et al., 2021), teaching replications in social sciences (Reedy et al., 2021), or leading a participant-guided ‘learn-by-doing’ course in meta-research, in which a multidisciplinary team of ECRs from different universities works together to design, conduct, and publish a meta-research, or ‘research on research’, study (Weissgerber, 2021). Resources for those interested in adopting this approach include methods for running these courses (e.g. Neuendorf et al., 2022; Weissgerber, 2021) and studies performed by course participants (Jambor et al., 2021; Kroon et al., 2022; Seibold et al., 2021). An alternative approach is to have undergraduate students conduct direct or conceptual replications as thesis projects (Jekel et al., 2020).

As a research group-based alternative to this approach, research group leaders or project supervisors can provide hands-on training in implementing reproducible research and open science practices in ongoing projects. Another approach is to complete a collaborative thesis. Here, undergraduate students from different universities collaborate on one project to increase sample size and statistical power (Button, 2018; Button et al., 2020). In these cases, reproducible research and open science practices may be applied while conducting traditional research, as opposed to meta-research or replication studies.

### Build communities

#### Strategy 9: Organize journal clubs and other community-learning opportunities

Community meetings can be easy to join and help participants gain knowledge on open science and reproducible research practices, while building a network. Formats include journal clubs, open science meetups or working groups, hacky hours, coding clubs, community-driven projects (e.g. Gierend et al., 2023), open science pedagogical communities and communities of practice. Journal clubs and other community activities are often organized by ECRs, for ECRs. Some of these formats can be implemented with a basic understanding of reproducible research and open science practices and require comparatively little infrastructure. Researchers can also incorporate materials on reproducible research and open science in existing journal clubs, meetups or working groups.

There are many examples of initiatives that offer community-learning opportunities; we recommend searching for initiatives that align with one’s interests and desired format. Organizations such as ReproducibiliTea help scientists set up local journal clubs by providing reading lists and instructions on how to start and run a journal club (Orben, 2019; Orben et al., 2018). This model has been used to establish over 100 journal clubs in 22 countries, as of February 2023. The Framework for Open and Reproducible Research Training (FORRT) pedagogical community facilitates collaborative development of educational materials (Armeni et al., 2021; Azevedo et al., 2022), provides a starting point for adopting improved research and pedagogical practices (Pownall et al., 2021) and offers a supportive environment for scholars to share experiences and lessons learned (Elsherif et al., 2022; Pownall et al., 2023).

#### Strategy 10: Create resource hubs

Resource hubs focusing on reproducibility and open science can be excellent tools to advocate for these practices while building communities. Resource hubs can serve numerous functions. For example, they can be a central hub for collecting resources, or providing training and consulting services for an institution or network. Hubs can also coordinate data collection and benchmarking activities, such as launching a survey to understand existing practices at an institution. Additionally, resource hubs can strengthen local science improvement communities by helping to implement other strategies described above.

Resource hubs include Open Science Centers, Open Science Offices, and Open Science Labs. An Open Science Office or Center might simply be a person or a small team with several paid hours a week devoted to organizing local activities for reproducible and open science practices. One example is the Open Science Office at the University of Mannheim, which includes an Open Science Officer and an Open Access Librarian. Their activities include organizing open science days and workshops, offering grants for open science projects, and providing infrastructure.

Some German institutions, departments, and libraries have established larger Open Science Centers, where personnel promote and foster reproducible and open science practices by offering education and training (such as the Leibniz-Institut für Psychologie), or by forming networks and communities of researchers (such as at LMU Munich and the University of Bielefeld; Hachmeister and Schirrwagen, 2021). The QUEST Center at the Berlin Institute of Health and Charité Universitätsmedizin – Berlin provides services to support reproducible research practices in the institutional community, while also conducting meta-research and serving as a test incubator for interventions to improve research (Drude et al., 2022; Strech et al., 2020).

Open Science Labs may work on open science research projects, creating and providing software, and organizing book sprints and hackathons (such as the Open Science Lab at the TIB at Leibniz Universitat Hannover).

An alternative approach is to create or contribute to decentralized online resource centers. These online communities are often run by volunteers and provide education and training on reproducible and open science practices. This may include curated databases of reproducible and open science-related resources, which are useful when setting up education and training programs. Several excellent online resource centers already exist, such as FORRT and the STEM Education Hub, which collaborates with the Center for Open Science.

#### Strategy 11: Connect individuals and initiatives involved in reproducible research and open science practices

Our virtual brainstorming event highlighted the need for individuals and organizations to connect those working on similar topics, or in the same institution or region. There were several cases where attendees at the same institution, or in the same region, had never met. Many attendees felt isolated with their activities. Connections between groups can facilitate collaborations, provide opportunities for shared problem solving and mentorship, and allow different groups to support and amplify each other’s efforts. Sharing materials and resources within collaborations might also lessen the workload for individuals. Collaborations allow groups to work across departments and fields, facilitating broader change within the institution. National reproducibility networks, like the GRN or UKRN (UK Reproducibility Network Steering Committee, 2021), and their local nodes, or the Network of Open Science Initiatives (NOSI) (Gebauer et al., 2017) may provide infrastructure and serve as ‘connectors’.

## Supporting activities: Allocate resources and monitor impact

As research institutions or individuals within institutions implement strategies to encourage responsible research and open science practices, it is important to allocate resources to support this work and monitor the impact of strategies that have been implemented.

### Allocate resources

The available resources constrain how each strategy can be implemented. All strategies require personnel time. In some cases, staff are already available and costs can be absorbed by prioritizing implementation of one or more of the eleven strategies described above. In other cases, additional funding and personnel may be needed. Creating resource hubs may be particularly resource intensive, as institutional open science centers or offices require dedicated staff and project funds. Courses and workshops are often offered by ECRs, for ECRs, on a volunteer basis. The time that ECRs invest in these programs is a common externalized cost. Institutions that cannot provide salary support for the time spent on these activities may be able to create small grants to cover incidental workshop costs, such as food, speaker fees or room booking fees. Institutions can also set up Open Science prizes or grants to further reward and incentivize open science and reproducible research practices. Institutions may also consider using some of the funding allocated for publication fees to collaboratively support non-commercial open science infrastructure (https://scoss.org/what-is-scoss/), including open access journals, preprint servers, and repositories for protocols, data, code, and other research products.

Research institutions lack the time, resources and expertise to implement all strategies. Starting with one or two achievable strategies helps to build awareness and skills within the institutional research community.

### Monitor impact

Monitoring provides institutional leadership with valuable insight into whether the strategies implemented within the institution are changing research culture and practice. Early-stage monitoring to assess participation in training or community building activities may be performed by those running courses or other activities. To assess impact, institutions can examine baseline rates of reproducible research and open science practices and changes over time as new strategies are implemented. Those involved in implementing various strategies can use this information to refine and adapt their programs, as well as the strategic plan for the institution. Impact may not be visible for many years, depending on the practice. Initiatives should not be discontinued if they fail to demonstrate immediate impact.

Institutional dashboards (such as the Charité dashboards for responsible research and clinical transparency) can rapidly disseminate information about reproducible research and open science practices within the institution (Cobey et al., 2023; Franzen and Salholz-Hillel, 2022). Another example of a monitoring activity includes the NeuroCure cluster of excellence, where staff monitor the percentage of papers that are open access and offer to deposit paywalled papers in open access repositories on authors’ behalf (Personal communication, René Bernard). A focus group on self-archiving has been established to connect personnel who offer self-archiving services at various institutions. Investigators at the Berlin Institute of Health at Charité Universitätsmedizin – Berlin received report cards outlining opportunities to improve transparency and reporting of timely results in clinical trials that they had conducted. This activity combines monitoring with an intervention to improve reporting.

Combining monitoring with research can provide further insight into strategies for running impactful programs. Research examining the impact of educational programs or changes to research assessment criteria, for example, may help to refine and assess the value of these programs. High quality studies in this area are unfortunately sparse, but there are first indications that implementing open science into teaching in higher education is beneficial for skill acquisition, and student engagement and satisfaction (Pownall et al., 2023). In addition to standard course evaluations, follow-up assessments for training programs may examine whether past participants implemented the practices taught in their own research projects. Understanding which practices participants are, and are not, implementing, and why, may help instructors to develop more effective programs. Furthermore, this information can be used to advocate for the widespread integration of reproducible research and open science training across institutes and universities and help inform institutional leadership about what types of resources, rewards, and incentives are needed to do so.

## Selecting strategies and tips for implementing strategies

Evidence on which of the strategies described above are most effective and impactful is lacking. Furthermore, each strategy can be implemented in many different ways, which may alter the impact. The authors propose that adapting research assessment criteria and program requirements may be the most impactful action that institutions can implement. Researchers have limited time and resources and many competing demands. If we want all investigators to prioritize reproducible research and open science practices, we need to normalize, reward, and incentivize these practices by integrating them into program requirements and research assessment criteria. Additionally, these strategies have the potential to reach researchers throughout the institution, extending beyond those who voluntarily attend trainings or join communities. Training materials on how to implement policy change (Kingsley and Shreeves, 2021) are particularly valuable for those working to reform research(er) assessment. Unfortunately, reforming research assessment criteria and program requirements may not be possible at institutions where support of institutional, departmental, or program leadership is lacking.

This potential impact of reforming research assessment criteria and program requirements should not detract from the importance of strategies focusing on offering training and building communities. This foundational work is critical to raise awareness and build capacity for making reproducible research and open science training the norm at research institutions. At some institutions, building communities and offering training may create a critical mass of advocates needed to convince institutional decision makers that changes to research assessment criteria and program requirements are needed. In addition to potential impact, individuals should consider their position and skills, available resources, and the constraints and opportunities introduced by the surrounding environment when selecting a strategy to work on.

This section offers tips for implementing strategies 4, 6, and 8; tips for implementing the other eight strategies are available in the .

### Tips for implementing strategy 4: Offer reproducible research and open science courses

#### Select appropriate course formats and topics

When organizing a course or training event, select formats that align with your expertise, available resources, and the amount of time that you can invest. Investigators with expertise on a particular topic, for example, may offer single lectures or webinars, or collaborate with others to offer a course or webinar series.

#### Join training programs

Offering a reproducible research and open science course can be overwhelming for new instructors. Join multidisciplinary training programs, such as Reproducibility for Everyone (https://www.repro4everyone.org/), or participate in train-the-trainer programs, as for example offered by the Carpentries (https://carpentries.org/), to gain experience.

#### Participate in team teaching

Team teaching is especially valuable when training covers many topics or is intended for a multidisciplinary audience. Instructors may specialize in different topics (e.g. data management vs. reporting guidelines), fields, or study types (e.g. in vitro vs. preclinical vs. clinical biomedical research). Consider sharing course syllabi and materials as open access resources via repositories (e.g. Zenodo, Open Science Framework, PsychArchives) to help make reproducibility and open science courses the norm.

#### Offer training to different audiences

Consider offering training at many different levels (e.g. individual researchers, research groups, departments, institutions) and for individuals at different career stages. Partner with different organizations (e.g. institutional training, conference workshops, trainings offered by scientific societies) to extend your reach.

#### Include interdisciplinary perspectives

The concepts and skills discussed in reproducibility and open science training typically apply to many fields. Participants benefit from learning how problems manifest across fields, and exploring solutions from other fields that may be adapted to their own work.

#### Reuse available (online) resources and adapt materials where needed

Before creating new resources, consult available online resources, such as open lesson plans, presentations, videos and interactive exercises (e.g., The Turing Way Community, 2022; https://www.oercommons.org/ for resources). Materials for multidisciplinary audiences can often be adapted by selecting the topics most relevant to a specific field or replacing general examples with field-specific alternatives. Expertise and resources can also be shared among colleagues within a research institution via ‘lessons learned’ or ‘best practices’ discussions (https://journals.qucosa.de/ll/index).

#### Consider administrative and organizational aspects

Course organization involves more than selecting the format and delivering content. You may need to advertise the event, invite participants, set up a registration site, organize a venue, make technical arrangements, and send out reminders. Institutions can support course organizers by providing resources or co-organizing larger courses.

### Tips for implementing strategy 6: Provide hands-on training

**Figure 2.**
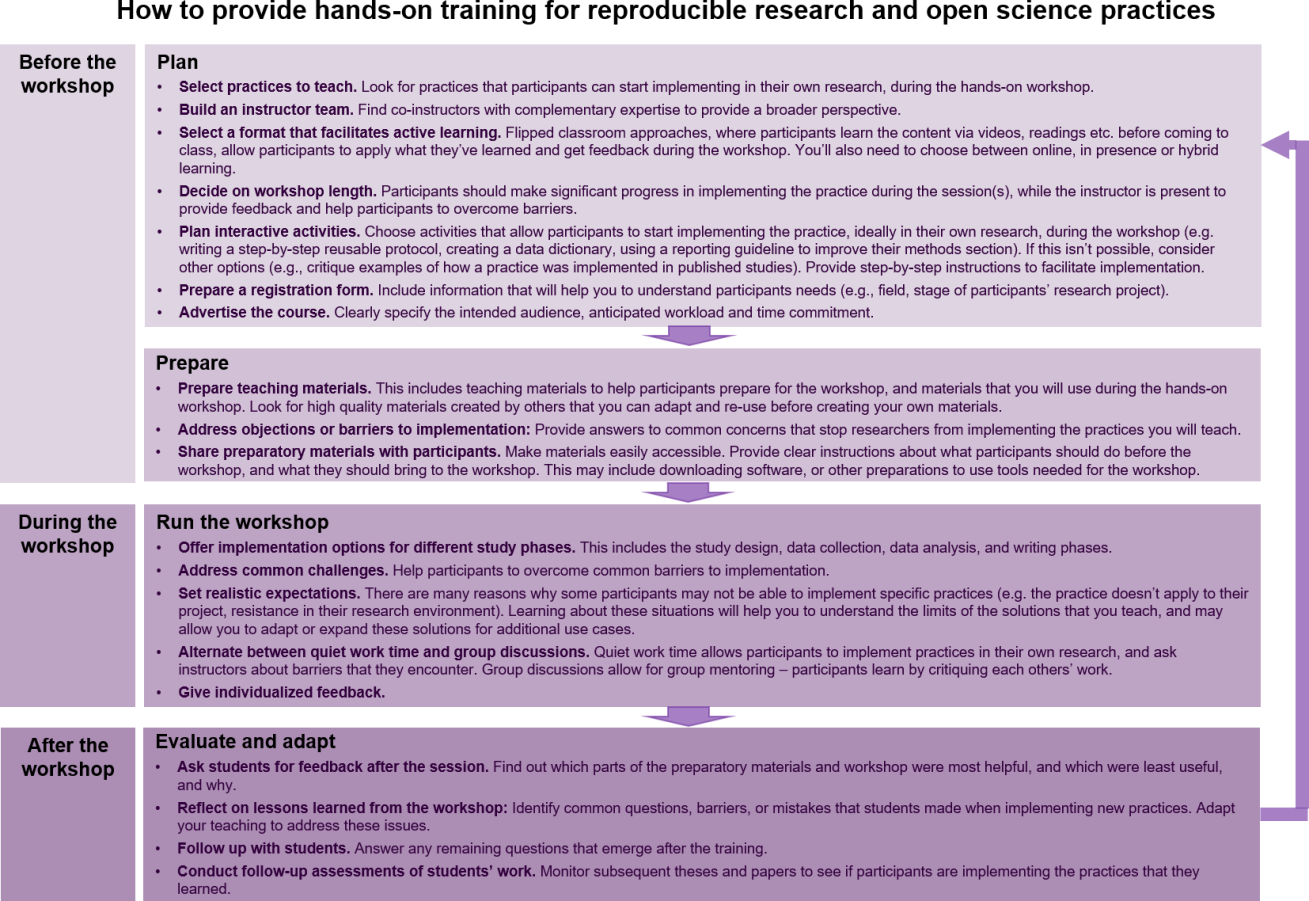
How to provide hands-on training for reproducible research and open science practices. The figure illustrates important points to consider before, during and after the training. Feedback and lessons learned from each training should be used to improve the next training session.

Figure 2 provides a detailed overview of the process for implementing Strategy 6.

#### Provide step-by-step instructions and feedback

This helps participants navigate early roadblocks, reducing barriers to implementation. Participants in hands-on courses also learn from each other’s questions and experiences.

#### Consider team teaching

While practical experience often increases the students’ motivation and confidence more than theoretical knowledge, teaching hands-on courses can be more challenging than giving straight lectures. Team teaching allows instructors to answer a broader range of questions, especially when participants come from different disciplines or have different study designs.

#### Clearly specify the intended audience, anticipated workload and time commitment in course advertisements

State the level of research and/or open science experience, the relevant fields or research designs, as well as the learning goals in the course announcement. This allows participants to select courses that teach skills relevant to their research. Explain any additional workload beyond the planned course time (e.g. preparatory tasks or homework) so that participants can plan accordingly.

#### Offer implementation options for different study phases

Address the different ways in which a skill might be implemented, depending on the phase of the participants’ research project (e.g. study design, data collection, data analysis, manuscript preparation). For example, creating a data management plan is most useful in the study design phase (Michener, 2015), while research resource identifiers (https://scicrunch.org/resources) can be added at any time.

#### Set realistic expectations for implementation

Emphasize that few research groups have the time and resources to implement all reproducible research practices simultaneously and participants may not be able to implement all practices in their day-to-day research. The practice may not apply to the participant’s research area or study phase, there may be obstacles to implementing the practice in the participant’s research project, or the participant’s advisors or co-authors may resist certain practices. Highlight the potential for ‘reverse mentoring’, where participants can serve as mentors to their own supervisors on specific topics (Pizzolato and Dierickx, 2022). Prepare participants to address common concerns or barriers that may be raised by co-authors (Heise et al., 2023).

### Tips for implementing strategy 8: Perform replication or meta-research studies as course projects

#### Consider the educational goals of the course, available resources, and student experience when designing a project

When conducting replication studies, for example, the project could focus on studies with open data and materials, or include studies with closed data and materials. Alternatively, instructors could contact the authors of studies to be replicated in advance to confirm that they can obtain data or support from the study authors (see, e.g. this video on learning good research practices the hard way). Whereas replicating studies with open materials may reduce students’ workload and reveal the advantages of open science, replicating studies without open materials teaches students about the importance of detailed methods. Students may also be involved in designing the project.

#### Carefully define the scope of the project

Participants should be able to complete the project with the time and resources available. Research projects can be predefined by course instructors or developed by participants in collaboration with the instructors. Project development is time-consuming and should be reserved for longer, more advanced courses (Weissgerber, 2021).

#### Ensure that you have adequate support

Courses where participants work together to complete a single research project are uniquely challenging for instructors, who must balance the project demands with constraints imposed by the course duration. Having a student assistant, who provides administrative support while doing the research project alongside participants, reduces instructor burden while providing training for the supporting student.

#### Integrate reproducible research and open science practices

This might include preregistration, protocol sharing, open data, open code, posting preprints, using ORCIDs and CRediT authorship statements, or many other practices.

#### Focus on why

During class discussions, encourage participants to identify different approaches that they might use to handle a particular aspect of the project, compare the strengths and weaknesses of those approaches, and retrospectively reflect on the impact of the approaches that they decided upon. Understanding why the class selected a particular approach for a specific situation teaches participants to implement theoretical principles within the constraints imposed by an actual research study.

#### Use unanticipated challenges as opportunities to teach problem solving skills

Unanticipated challenges occur in every research project. They provide students with an opportunity to adapt and apply what they have learned by balancing theoretical principles with real-world constraints.

#### Create a positive and inclusive team dynamic

Ensuring that all team members are comfortable sharing ideas is essential for collaboration. Discuss strategies for good communication in multidisciplinary and multicultural teams. Encourage participants to get to know one another, work in small groups and take advantage of leadership opportunities. We encourage readers to consult additional resources on these important topics.

#### Plan ahead if you aim to publish the study

Rigorous design is critical for publication. Establish transparent strategies for allowing the class to determine authorship order. Use CRediT (https://credit.niso.org/) and/or MeRIT (Nakagawa et al., 2023) authorship statements to report participant contributions. Carefully explain each stage of the publication process for students who have limited experience with publishing. Stay in contact with participants until the manuscript is published.

## Limitations

Several limitations of the present work and the virtual brainstorming event have to be considered. All participants were working in Germany. Many of them worked in psychology or the biomedical sciences. The strategies shared may not be generalizable to other fields or countries. Integrating additional fields into the discussion is important to facilitate systemic change that meets the needs of departments throughout the institution. Further, most participants were working on grassroots activities. Crucial infrastructure personnel, such as librarians or software engineers, were underrepresented. Exploration of top-down strategies for making reproducible research and open science training the norm is needed. This will require other stakeholders, particularly those in leadership or administrative positions. While this paper offers tips and lessons learned based on participants’ experiences, it is not a qualitative research study. Studies examining whether the practices discussed increase the proportion of research that implements reproducible research and open science practices are needed. The proposed approaches may not be feasible for all institutions, departments or research fields, or may need to be adapted to meet local needs.

## Conclusions

The eleven strategies discussed here highlight that there are several actions that can be taken to make reproducible research and open science training the norm at research institutions. Many of these strategies go beyond offering courses and workshops on these topics. Researchers can take action in their roles as scientists, supervisors, mentors, instructors, and members of curriculum design or hiring and evaluation committees. Combining these bottom-up activities with top-down efforts by institutional leadership and research support staff (including librarians, information technology professionals, and members of administrative committees) could accelerate institutional implementation of reproducible research and open science practices across disciplines. Research institutions can further support these activities by allocating resources and monitoring participation and impact. Sharing expertise among institutions may also be beneficial. Making reproducible research and open science training the norm will require a broad coalition, and we hope that this piece will inspire others to join these efforts.

## Additional files

Supplementary file 1. Event format and tips for implementing strategies (1–3, 5, 7 9–11).
