# Supplementary material for: Eleven strategies for making reproducible research and open science training the norm at research institutions
Source: eLife. 2023 Nov 23;12:e89736. doi: 10.7554/eLife.89736 (PMC10666927; doi:10.7554/eLife.89736)
Supplement: Supplementary file 1. [file elife-89736-supp1.docx]

**Kohrs et al. 2023. Eleven strategies for making reproducible research and open science training the norm at research institutions. eLife 12:e89736**

**Supplementary file 1: Event format and tips for implementing strategies (1–3, 5, 7 9–11)**

**Event format**

**Methodological details for the virtual brainstorming event**

The organizers (SA, ABB, SF, TH, VH, CH, TLW) invited participants to attend the brainstorming event “Making reproducibility and open science education and training the norm” in March 2022. Participants were invited by contacting nodes of the German Reproducibility Network, and disseminating information through mailing lists and organizations focused on reproducible research and open science training in Germany. The virtual brainstorming consisted of (1) a virtual networking event prior to the brainstorm, (2) an asynchronous virtual brainstorming using an online discussion board, and (3) small, live group discussions in virtual meetings on various topics related to making reproducible research and open science education and training the norm. The format used for this event has been described in detail previously (Holman et al., 2021).

The brainstorming event included the organizers and 96 active participants over two days. The organizers used guiding questions to structure the asynchronous discussion:

1. What resources and strategies are already being used to train researchers in reproducibility and open science in Germany?
2. What opportunities and creative solutions exist to expand and improve reproducibility education in Germany?
3. How can we make reproducibility education/training the norm in Germany? What top-down and bottom-up approaches can we use? What are the barriers we face and how can we overcome them to make reproducibility education and training the norm?

Discussions included small group conversations in virtual meetings and asynchronous written conversations on the online platform Slack. Event organizers later synthesized these discussions into the eleven strategies and tips presented in this paper. No formal methodological procedure was used to formulate the outlined strategies.

An outline and several draft versions were shared with event participants to get their feedback and the manuscript was adjusted accordingly. Participants who provided input to the outline and manuscript draft were included as co-authors.

#

# **Tips for implementing strategies (1–3, 5, 7 9–11)**

## There are several tips that potentially apply to the first three strategies (which are all related to adapting research(er) assessment criteria and program requirements

## .

**Use leadership positions:** Leadership positions offer opportunities to facilitate widespread changes in policy and practice. If you hold a leadership position, explore opportunities to implement systemic changes to improve research culture and practice.

**Learn from successful examples:** Talk to others at institutions who have successfully implemented changes that you would like to introduce at your institution. Ask them to share tips, tricks and lessons learned, along with policies or other documentation.

**Know the process for policy change at your institution:** Consult resources on how to change an implement policies at research institutions (e.g. Kingsley and Shreeves, 2021). Next, find out how to change policies at your institution. This includes determining what individuals or committees must approve policy changes, determining who sits on these committees and when they meet, and how to get something on the agenda.

**Tips for implementing strategy 1: Adjust curriculum to require reproducibility and open science training**

**Be persistent**: Curriculum change is time consuming and requires top-down and bottom-up approaches, including support from institutional decision makers. Collaborate with administrators and curriculum committee members to add a new course to the curriculum or to make a course mandatory that was previously offered as an elective. If needed, repeat this process with committees from different departments and programs, adapting the course content to the program’s needs.

**Anticipate resistance**: You may encounter resistance to adjusting the curriculum. Prepare responses to common concerns. Advocate for mandatory reproducibility and open science courses by using funding agency and journal mandates, information on the prevalence of these practices in the field, examples highlighting the problem of non-reproducible research within the field, course outlines and student feedback from existing courses.

**Seize opportunities**: Stay in contact with administrators. While rare, the addition of new degree programs or restructuring of existing programs offers an excellent opportunity to require reproducible research and open science courses.

**Tips for implementing strategy 2: Require reproducible research and open science practices in undergraduate or graduate theses**

**Establish systems that encourage students to integrate reproducible research and open science practices early**: Listing required practices (e.g., pre-registration of the thesis or thesis chapters, open code in form of a codebook or reproducible script, open materials) in the thesis agreement ensures that students are exposed to these concepts before starting their research. They can work with supervisors to develop plans to integrate reproducible and open-science-related practices from the beginning. Students gain hands-on experience with the challenges and benefits of applying these practices in their own work (Kathawalla et al., 2021).

**Form your own agreement**: If your institution or department does not have requirements for reproducible research and open science practices in graduate theses, form your own individual agreement. List applicable practices and, if possible, describe how these will be part of the evaluation process. This can be initiated by the supervisor or by the supervised student.

**Assess thesis requirements continuously**: When adding new criteria, re-evaluate all degree requirements to ensure that they incentivize responsible research practices, are feasible, and can be completed in the expected timeframe. Reproducible and open science practices should be rewarded during thesis evaluation (e.g., by integrating them into the grading process), as they increase student workload.

**Make additional materials available if possible**: Including reproducible and open science practices into undergraduate and graduate theses leads to the generation of additional materials (e.g., thesis pre-registration, methods, data and code). Consider depositing these outputs on public repositories alongside submitting these privately along with the thesis.

**Tips for implementing strategy 3: Include reproducible and open science practices in research(er) assessment**

**Consult existing resources and adapt where needed**: A task force established by the Deutsche Gesellschaft für Psychology (German Psychological Society, DPG) works on overarching guidelines to incorporate reproducible and open science practices in hiring and promotion procedures in psychology (Gärtner et al., 2023, 2022; Leising et al., 2022; Schönbrodt et al., 2022). The concrete suggestions provided may be adapted to selection processes in other scientific fields.

**Provide assessment guidelines**: Specify procedures for assessing and scoring open science indicators and reproducible research practices, and describe these procedures in materials for those being evaluated. Incorporate these procedures into an assessment guideline to share with hiring and evaluation committee members. This approach is currently implemented by the Berlin Institute of Health at Charité Universitätsmedizin - Berlin (<https://www.bihealth.org/en/notices/wt-bih-merit>, Kip et al., 2022a, 2022b).

**Involve non-committee members in the process**: Support committee members in applying responsible research indicators by including a non-voting member who is an expert in evaluating reproducible research and open science practices, as is done by the Berlin Institute of Health and Charité. Further, include early career researchers (ECRs) in the discussion about research assessment, as their perspectives can be helpful in adapting current criteria to incentivize responsible practices. ECRs often champion these efforts and some have specialized training in new approaches and tools for reproducible research and open science.

**Allow for a transition period**: When adjusting research(er) assessment, notify all stakeholders involved in advance and allow for a transition period. This helps to avoid placing those (early career) researchers at a disadvantage who were originally working under different assessment guidelines.

**Tips for implementing strategy 5: Integrate reproducibility and open science skills into courses on other topics**

**Incorporate hands-on experience and real-life examples**: Incorporate skills into practical or laboratory sessions to provide students with hands-on experience. This is especially valuable for undergraduate students who are not yet conducting their own research. Share examples of how a particular practice has enhanced or harmed research in the student’s field to highlight the relevance of practices that you discuss.

**Collaborate across the curriculum**: Work with colleagues who teach subsequent courses to reinforce and build upon reproducible research and open science skills. Contact support staff (e.g., curriculum committee members, or program coordinators) to integrate training opportunities throughout the curriculum (see also Strategy 6).

**Tips for implementing strategy 7: Conduct educational interventions for research groups**

Group interventions may be very different depending on the context and the practice being implemented. While this strategy has great potential, it was one of the least common ones amongst event participants. More sharing of experiences on how to effectively implement this strategy is needed.

**Ensure that the team leader is supportive:** The work environment plays an important role in enabling teams to implement new practices, on both structural and social levels. Work with the team leader to address concerns and confirm their commitment to the proposed changes before starting the intervention.

**Include everyone**: Involve the whole research team when implementing new practices as this allows members to share expertise, identify common goals, and decide on reusable tools and procedures. Discuss concerns and barriers openly and transparently within the group.

**Tips for implementing strategy 9: Organize journal clubs and other community-learning opportunities**:

**Foster accessible discussions**: Making discussions accessible to everyone can be challenging, as some participants may have extensive knowledge of reproducibility and open science topics, whereas others may have no prior experience. This can lead to attrition. Consider running “beginner” and “advanced” community meetings or assigning more experienced mentors to beginners. Provide a list of key publications or resources already discussed in previous sessions (e.g., Wessolowski et al., 2021). Assign different roles to individuals in the meetings and discussions depending on their interests and expertise (e.g., discussion facilitator, meeting organizer, presenter introducing the topic of the meeting to the community, critic).

**Build communities**: Organize regular meetings to make it easy for participants to engage with others who are interested in reproducible and open science practices. Informal formats, such as open science lunches, can be useful here. Depending on expertise, use hacky hours or other formats to create a community around open code, tools and new techniques, and discuss practical considerations for implementing these techniques. Materials such as the Open Science Community Starter Kit from the International Network of Open Science/Scholarship Communities can help you get started (<https://www.startyourosc.com/>).

**Share information and resources**: Keeping your community members informed and attracting new participants are challenging, but important, tasks. Consider using different formats (e.g., talks, workshops, position papers) and platforms (e.g., online discussion forums, wikis, repositories) to engage with others while sharing materials and updates.

**Adjust the frequency of meetings to meet your community's needs:** If meetings take place too often, attendance may decrease. Clarifying which frequency works for the community (e.g., once per term) helps to ensure attendance and efficient time use.

**Tips for implementing strategy 10: Create resource hubs**

**Start with an attainable goal**: Resource hubs range from small teams to centers with extensive resources and infrastructure. Before planning and setting up a resource hub, identify institutional or administrative support and consider the resources available to you. Starting with a smaller hub or team allows you to gain experience, build momentum, and refine your approach and activities. Once the hub is established, you can amplify your efforts and expand.

**Identify allies and collaborators**: Contact individuals, offices or centers who are organizing activities that you would like to establish in your research hub environment. Build a network of collaborators who can support you in creating or amplifying a resource hub. In addition, seek out the support of institutional leadership and administrative staff. These are important allies when creating new resource hubs.

**Consider funding and sustainability**: The amount and source of funding, as well as any conditions for renewal, will influence the scope and priorities of the hub. Short-term funding may require staff to focus on rapidly achievable goals that add value to the community, whereas longer-term funding may allow staff to address more complex topics.

**Tips for implementing strategy 11: Connect individuals and initiatives involved in reproducible research and open science practices**

**Seek out networking opportunities**: Participate in networking events, such as our virtual brainstorming event, to identify others that share your interests regarding reproducible and open science training, learn from each other, and join ongoing activities. Alternatively, organize an event yourself.

**Identify external support**: To achieve your goals, you may need expertise that is not available locally. Use living “speaker directory” documents to identify speakers, collaborators or others with relevant expertise (e.g., <https://www.ukrn.org/speaker-directory/>).

**Connect with supporters to obtain feedback**: Identify and communicate with individuals who are not actively involved in reproducible research and open science, but who support these practices and can give constructive feedback. Feedback can help you to assess the feasibility of proposed practices, while identifying and addressing barriers to implementation.

**Consult experts when necessary**: Sometimes issues arise that require specific expertise (e.g. seeking legal advice when dealing with intellectual property issues). Consult appropriate experts or departments to reduce risk and facilitate the implementation.

**References**

Gärtner A, Leising D, Schönbrodt FD. 2023. Empfehlungen zur Bewertung wissenschaftlicher Leistungen bei Berufungsverfahren in der Psychologie. *Psychologische Rundschau* **74**:166–174. doi:10.1026/0033-3042/a000630

Gärtner A, Leising D, Schönbrodt FD. 2022. Responsible Research Assessment II: A specific proposal for hiring and promotion in psychology (preprint). PsyArXiv. doi:10.31234/osf.io/5yexm

Holman C, Kent BA, Weissgerber TL. 2021. How to connect academics around the globe by organizing an asynchronous virtual unconference. *Wellcome Open Res* **6**:156. doi:10.12688/wellcomeopenres.16893.2

Kathawalla U-K, Silverstein P, Syed M. 2021. Easing Into Open Science: A Guide for Graduate Students and Their Advisors. *Collabra: Psychology* **7**:18684. doi:10.1525/collabra.18684

Kingsley D, Shreeves S. 2021. OSF | T15: How to introduce and implement policy in your institution and still have friends afterwards. https://osf.io/7whxe/

Kip M, Dirnagl U, Koenig S, Wiebach J, Dunkel M, Mallach M. 2022a. MERIT App Charité. https://osf.io/p2vyw/

Kip M, Koenig S, Dirnagl U. 2022b. Mechanisms of robust, innovative and translational research (MERIT). doi:10.17605/OSF.IO/ZMUHW

Leising D, Thielmann I, Glöckner A, Gärtner A, Schönbrodt F. 2022. Ten steps toward a better personality science – how quality may be rewarded more in research evaluation. *Personal Sci* **3**:e6029. doi:10.5964/ps.6029

Schönbrodt FD, Gärtner A, Frank M, Gollwitzer M, Ihle M, Mischkowski D, Phan LV, Schmitt M, Scheel AM, Schubert A-L, Steinberg U, Leising D. 2022. Responsible Research Assessment I: Implementing DORA for hiring and promotion in psychology (preprint). PsyArXiv. doi:10.31234/osf.io/rgh5b

Wessolowski N, Fricke K, Herrmann A, Wendt M, Tigges D, Niemann M, Mueller-Alcazar A, Lischke A, Vogel S, Ocklenburg S, Shedden-Mora M, Straßburger VM, Knabe N, Morgenroth O, Langkamp T, Kauff M, Friese U. 2021. Open Science Workgroup, Medical School Hamburg. doi:10.17605/OSF.IO/5SEZD
